# Chest ultrasound is better than CT in identifying septated effusion of patients with pleural disease

**DOI:** 10.1038/s41598-024-62807-4

**Published:** 2024-05-25

**Authors:** Linhui Yang, Kaige Wang, Weimin Li, Dan Liu

**Affiliations:** 1 Department of Respiratory and Critical Care Medicine, West China Hospital, Sichuan University, Chengdu, Sichuan China; 2 State Key Laboratory of Respiratory Health and Multimorbidity, West China Hospital, Sichuan University, Chengdu, Sichuan China

**Keywords:** Septated pleural effusion, Medical thoracoscopy, Ultrasound, Enhanced computer tomography, Tuberculosis, Respiratory tract diseases

## Abstract

Septated pleural effusion is very common. The presence of septations in pleural effusion determines the local treatment strategy for such patients. Therefore, there is a pressing need for imaging techniques to assess the presence of septations. The objective of this research was to assess the diagnostic efficacy of computed tomography (CT) and chest ultrasound in identifying septated pleural effusion. We delineated the ultrasound and enhanced chest CT manifestations for diagnosing septated pleural effusions, and subsequently, we conducted a comparative analysis to assess the diagnostic efficacy of enhanced chest CT and ultrasound in identifying septated pleural effusions. Medical thoracoscopy served as the gold standard for confirming the diagnosis of septated pleural effusions. Ultrasound demonstrated a sensitivity of 82.6% (95% CI 73.3–89.7%) and a specificity of 100.0% (95% CI 98.1–NaN) for diagnosing septated pleural effusion. In comparison, enhanced chest CT exhibited a sensitivity of 59.8% (95% CI 49.0–69.9%) and a specificity of 87.0% (95% CI 81.5–91.4%). The positive predictive value for ultrasound was 100.0% (95% CI 95.3–100.0%), while for enhanced chest CT, it was 68.8% (95% CI 59.0–77.4%). Ultrasound yielded a negative predictive value of 92.3% (95% CI 87.5–NaN), and enhanced chest CT had a negative predictive value of 82.0% (95% CI 74.6–87.8%) in diagnosing septated pleural effusion. Thoracic ultrasound exhibits superior sensitivity and specificity compared to enhanced chest CT in diagnosing septated pleural effusions. Therefore, chest ultrasound is highly recommended as an adjunct for determining septated pleural effusion.

## Introduction

The incidence of pleural effusion is on a steady rise each year^1–3^. Septated pleural effusion is very common, especially in patients with complex pleural effusion, tuberculous pleurisy, and malignant pleural effusion. Some studies have indicated that patients with malignant pleural effusions who exhibit a septated pleural effusion tend to have a worse prognosis^4^. The presence of septations in pleural effusion determines the local treatment strategy for such patients^3^. Therapeutic thoracentesis or tube thoracostomy alone does not seem to be sufficient for patients with septated parapneumonic effusions^5^. An article published in 2023 discussing the management of chest infections notes that due to the potential impact of nonuniform drug distribution within the septated space, current guidelines either remain neutral or advise against the utilization of intrathoracic antibiotics in cases of acute chest infections^3^, and fibrinolytics, VATS, and surgery are acceptable methods of managing patients with septated pleural effusions^5^. Therefore, there is a pressing need for imaging techniques to assess the presence of septations.

Although many study have suggested that multiple imaging examinations can identify the septations of pleural effusion^6–8^, there is no study confirming which method can better identify the septation of pleural effusion. The objective of this research was to assess the diagnostic efficacy of computed tomography (CT) and chest ultrasound in identifying septated pleural effusion. The findings aim to establish a reliable foundation for imaging evaluations, guiding decisions on invasive procedures such as pleural fluid drainage and intrathoracic fibrinolysis therapy of patients with pleural effusion.

## Methods

### Patient inclusion and exclusion criteria

**Figure 1:**
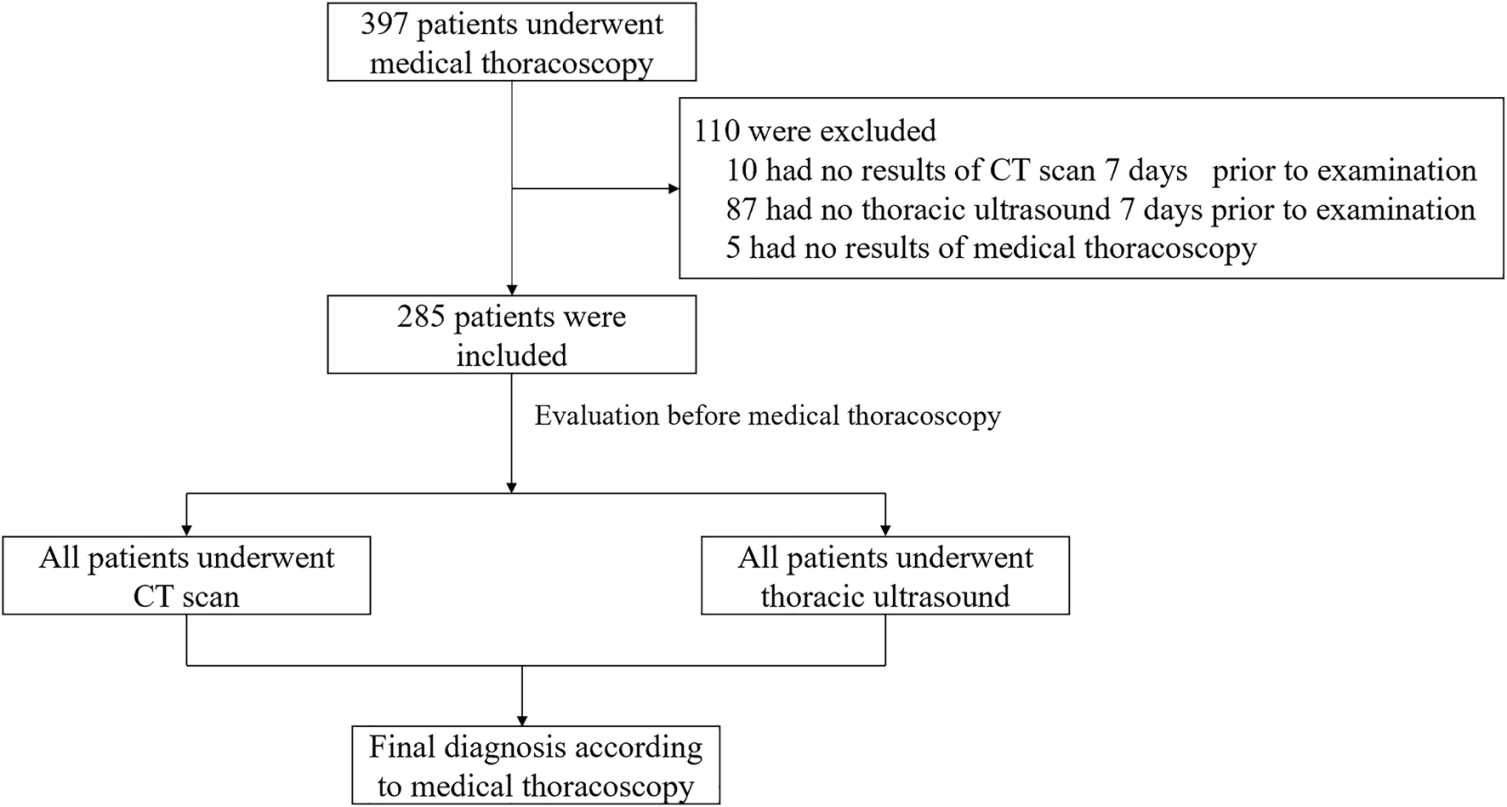
Flowchart for inclusion of patients.

A total of 397 patients diagnosed with pleural effusion, who underwent medical thoracoscopy at West China Hospital of Sichuan University within the last five years, were included in this study. Approval for the study was obtained from the Department of Clinical Research Administration at Sichuan University. Given the retrospective nature of the study, informed consent was waived for the included patients according to the Biomedical Ethics Review Committee of West China Hospital of Sichuan University. All methods were performed in accordance with the relevant guidelines and regulations. The exclusion criteria were as follows: (1) Lack of Enhanced Chest CT within 7 days before the medical thoracoscopy examination; (2) absence of chest ultrasound within 7 days before the medical thoracoscopy examination. (3) Unavailability of thoracoscopic images. Ultimately, 285 patients were included in the final analysis, as illustrated in Fig. 1. The medical thoracoscopic presentation of the pleural cavity served as the gold standard for diagnosing septated pleural effusion.

### Collection and interpretation of patient imaging information

**Figure 2:**
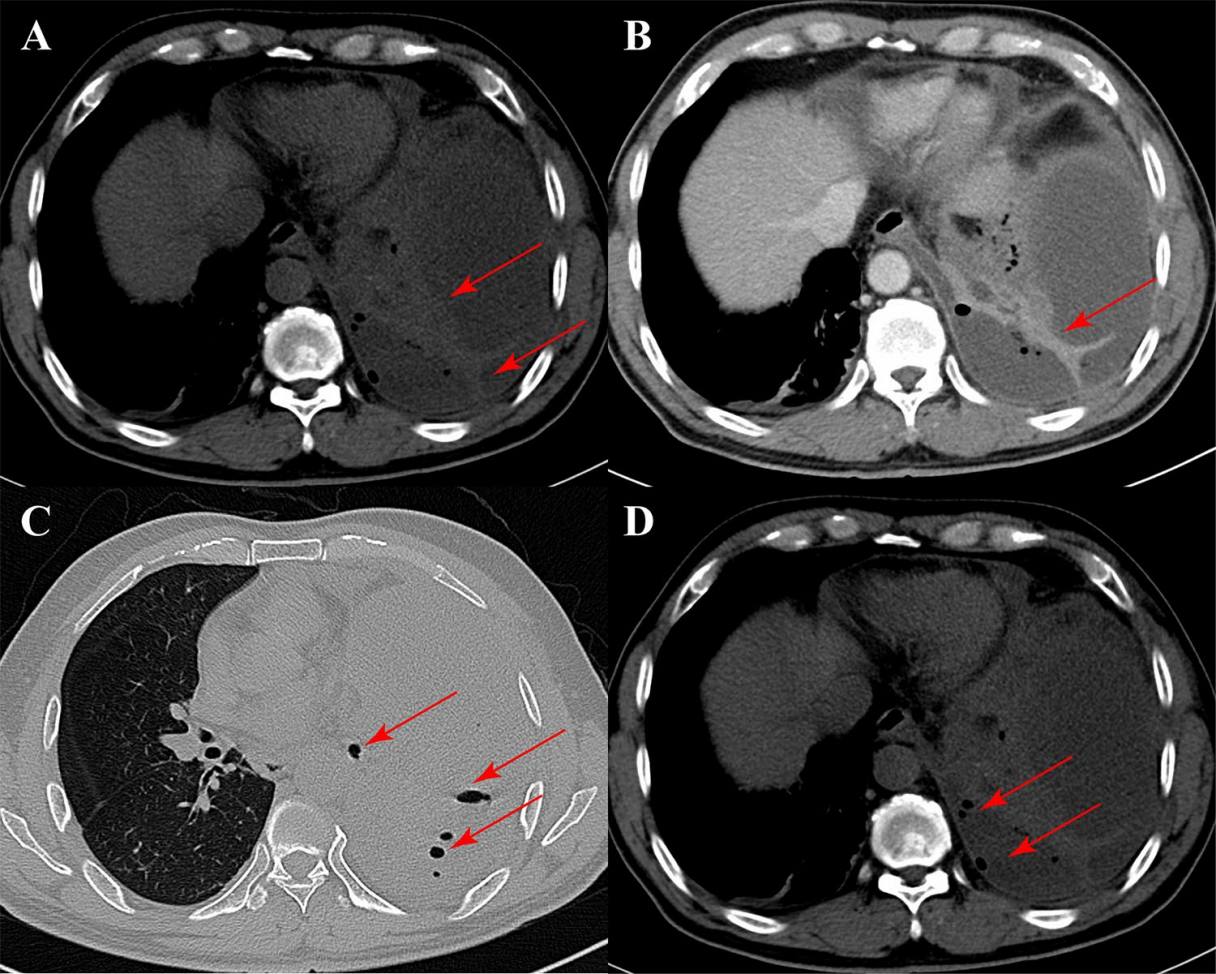
(**A**) Depiction of a septated pleural effusion on a plain CT scan, highlighted by the red arrow indicating the septum; (**B**) Illustration of a septated pleural effusion on an enhanced CT scan, highlighted by the red arrow indicating the septum; (**C**) Visualization of the bubble sign on a plain CT scan, featuring a red arrow indicating the bubble; (**D**) Display of the bubble sign on an enhanced CT scan, with a red arrow pointing to the bubble.

**Figure 3:**
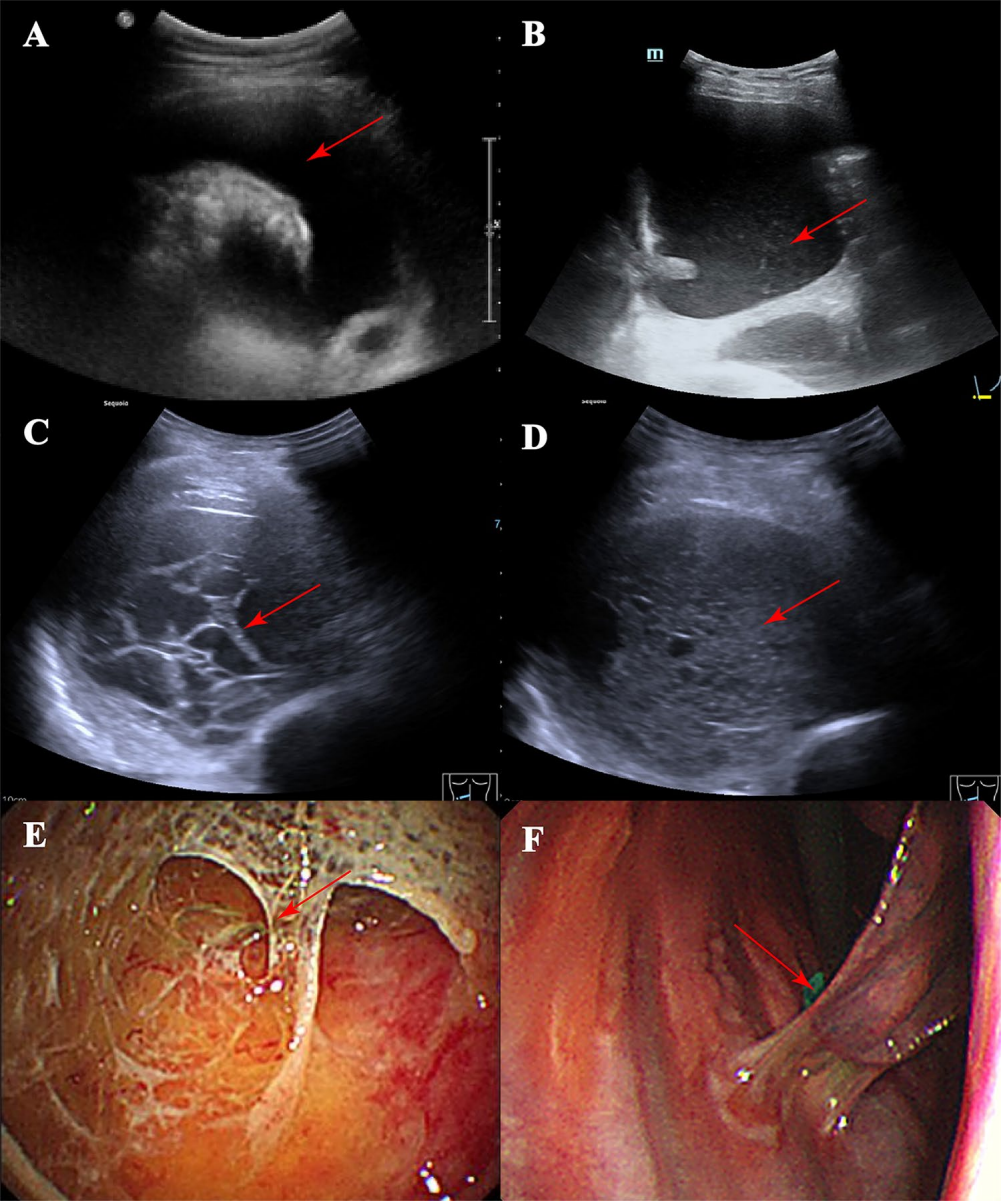
(**A**) Demonstration of anechoic regions on ultrasound imaging. (**B**) Visualization of complex yet non-septated areas on ultrasound. (**C**) Illustration of multiple septations on ultrasound. (**D**) Display of homogeneous complex strong echoes on ultrasound imaging. (**E**) Medical thoracoscopic presentation of a multilocular septation. (**F**) Medical thoracoscopic presentation of a small number of adhesions but no multilocular septations.

Clinical data including age, gender, serology, and imaging manifestations of pleural effusion were gathered from a cohort of 285 patients. To standardize the assessment of pleural effusion, distinct criteria were applied for both chest CT and ultrasound evaluations. In this study, for CT evaluation, three respiratory physicians with over 5 years of experience conducted the chest CT evaluations in this study. In instances of uncertainty, the three respiratory physicians engaged in discussion and reached a consensus through voting, selecting results favored by two or more of them. Similarly, ultrasound examinations of the patients were performed and evaluated by three ultrasonographers, each with over five years of experience. They utilized a convex array probe with a frequency of 3.5 MHz (gain 40–50 dB, depth 5–10 cm) to conduct the examination and recorded images and videos of the procedure. In case of uncertainty, the three ultrasonographers engaged in discussion and reached a consensus through voting, selecting results favored by two or more of them. For enhanced chest CT scans, effusions displaying septations (Fig. 2A,B), or the presence of multiple locules of air (Fig. 2C,D) within the effusion were classified as septations^6,9^. Conversely, in the absence of these discernible signs, the pleural effusion was categorized as non-separating. According to previous studies^7,10–12^, ultrasound characteristics were stratified into four distinctive types based on the presentation of pleural effusion: (1) Anechoic Areas (Fig. 3A): Anechoic (or non-echogenic) effusions are found when no ultrasound waves are reflected in the fluid and will look completely black on the screen, which means that there are no particulates in the effusion^11^. (2) Indicative of complex echogenicity without evident septation (Fig. 3B): Echogenic effusions demonstrate swirling or floating echoes which reflect the ultrasound waves, showing gray/white areas in the fluid. These echoes represent variable amounts of fibrin strands, protein, blood, or pus, which are moved by the cardiac pulsation^11^. (3) Complex Echogenicity with Visible Grid Septation (Fig. 3C): Septated effusions are characterized by the visualization of strands within the effusion; these can be free-floating and thin or more advanced and able to divide the effusion into discrete pockets^11^. (4) Markedly homogeneous enhancement of echogenicity (Fig. 3D): Demonstrating uniform and intense enhancement of echogenicity. In this study, the third ultrasound manifestation, characterized by complex echogenicity with visible grid septations, was adjudicated as indicative of a septated pleural effusion, while all other types were classified as non-septated pleural effusion. Medical thoracoscopic presentations further guided our classification, with individuals exhibiting multilocular septations considered to have septated pleural effusions^6^ (Fig. 3E), and all others categorized as non-septated pleural effusions. In other words, pleural effusions with only minimal adhesions in the pleural cavity that did not give rise to multiple compartments were excluded from the septated pleural effusion category (Fig. 3F). This rigorous adjudication process ensures accuracy and consistency in the characterization of pleural effusion patterns in our study.

### Statistical methodology

This study was statistically analyzed using R studio version 4.3.2. For normally distributed continuous variables, we used mean and standard deviation to describe the whole. And for skewed distribution variables, we used interquartile spacing to describe the whole. In addition, we described the diagnostic efficacy of ultrasound and chest CT for septated pleural effusion in terms of sensitivity, specificity, positive predictive value, and negative predictive value.

### Ethical approval and consent to participate

This study was approved by the Biomedical Ethics Review Committee of West China Hospital of Sichuan University. The informed consent was waived by the Biomedical Ethics Review Committee of West China Hospital of Sichuan University.

## Results

A total of 285 patients were included in the analysis of clinical features and imaging characteristics. Among them, 168 were male, constituting 59% of the total study population (Table 1). The average age of the patients was 56.5 years. Diagnostic categorizations revealed that 67 patients were ultimately diagnosed with infectious diseases, 108 with tumors, and 94 with tuberculous pleurisy. Notably, tumors represented the largest diagnostic category, accounting for 37.9% of the total cases. As part of ancillary tests pertaining to the nature of pleural effusion, 245 patients (86% of the cohort) exhibited exudative pleural effusions. Additionally, we conducted a follow-up on patient survival. Among the 161 patients with infectious pleural effusion (including tuberculosis and other infections), 25 were lost to follow-up, with 8 deaths in the no-septated group (78 cases in total) and 6 deaths in the septated (58 cases in total) group, and there was no statistically significant difference in prognosis between the two groups (P = 0.987); Among the 108 patients with malignant pleural effusion, 18 were lost to follow-up. In the non-septated group (70 cases), there were 31 deaths, whereas in the septated group (20 cases), there were 15 deaths, showing a statistically significant difference between the two groups (P = 0.022).

**Table 1:** Basic profile of 285 patients and disease types.

Clinical characteristic	
Male No. (%)	168 (59.0)
Age (year), mean ± SD	56.5 ± 13.9
Complementary examination	
WBC (10^9^/L), mean ± SD	7.0 ± 3.2
Albumin of blood (g/L), mean ± SD	36.1 ± 5.6
LDH (blood) (IU/L), median (Q1, Q3)	167.0(145.0, 200.0)
Number of nucleated cells in pleural fluid, median (Q1, Q3)	70.0(100.0, 1500.0)
ADA (pleural fluid), median (Q1, Q3)	15.5(8.6, 37.0)
Glucose (pleural fluid) (mmol/L), mean ± SD	4.9 ± 2.6
Albumin ratio, median (Q1, Q3)	0.7(0.6, 0.8)
LDH ratio, median (Q1, Q3)	1.8(1.1, 4.9)
Exudative pleural effusion No. (%)	245 (86.0)
Diagnosis	
Infection No. (%)	67 (23.5)
Tumor No. (%)	108 (37.9)
Tuberculosis No. (%)	94 (33.0)
Other diseases No. (%)	16 (5.6)

The comprehensive evaluation of 285 patients included an in-depth analysis of chest CT imaging features, chest ultrasound performance, and medical thoracoscopic findings, detailed in Table 2. In the realm of enhanced chest CT presentations, 59 patients exhibited septations of this subset, 39 (66.1%) displayed thoracoscopic evidence of septation, while 20 (33.0%) did not manifest septation during thoracoscopy. Additionally, 37 patients demonstrated multiple air bubble shadows on chest CT, with 28 (75.7%) exhibiting medical thoracoscopic indications of septation and 9 (24.3%) presenting without septation during thoracoscopy. In terms of ultrasound presentation, 179 patients displayed anechoic areas, of which 7 (3.9%) demonstrated multiple septations under medical thoracoscopy, while the vast majority (96.1%) exhibited no septations during medical thoracoscopy. Furthermore, 14 patients presented with a complex ultrasound pattern devoid of septation. Among them, only 1 (7.1%) showed multiple septations during medical thoracoscopy, while 13 (92.9%) showed no septation. A subgroup of 76 individuals exhibited both complex and septated ultrasound presentations, all of whom demonstrated multiple septations during thoracoscopy. Conversely, 16 patients with a homogeneous complex echogenic image on ultrasound displayed varied medical thoracoscopic outcomes: 8 (50%) exhibited septation, while the remaining 8 (50%) showed no septation during thoracoscopy.

**Table 2:** Comparison of different manifestations under ultrasound/chest CT with MT manifestations.

	Different types of presentation of hydrothorax	Separated in MT No. (%)	Non- separated in MT No. (%)	Add up the total
Ultrasound	Anechoic	7 (3.9)	172 (96.1)	179
	Complex non-separated	1 (7.1)	13 (92.9)	14
	Complex separated	76 (100.0)	0 (0.0)	76
	Homogeneously complex	8 (50.0)	8 (50.0)	16
Chest CT	Septations	39 (66.1)	20 (33.0)	59
	Multiple locules of air	28 (75.7)	9 (24.3)	37

We next analyzed the sensitivity, specificity, positive predictive value, and negative predictive value of enhanced chest CT and chest ultrasonography for determining septations of effusion, using medical thoracoscopic performance as the gold standard for diagnosing septations of effusion (Table 3). Table 4 describes the diagnostic efficacy of thoracic ultrasound and enhanced chest CT for septated pleural effusions. The sensitivity of ultrasound for the diagnosis of septated pleural effusion was 82.6% (95% CI 73.3–89.7%), whereas the sensitivity of enhanced chest CT for the diagnosis of septated pleural effusion was 59.8% (95% CI 49.0–69.9). The specificity of ultrasound for the diagnosis of septated pleural effusion was 100.0% (95% CI 98.1–NaN), whereas the specificity of enhanced chest CT for the diagnosis of septated pleural effusion was 87.0% (95% CI 81.5–91.4). The positive predictive value of ultrasound for the diagnosis of septated pleural effusion was 100.0% (95% CI 95.3–100.0), whereas the positive predictive value of enhanced chest CT for the diagnosis of septated pleural effusion was 68.8% (95% CI 59.0–77.4). The negative predictive value of ultrasound for the diagnosis of septated pleural effusion was 92.3% (95% CI 87.5–NaN), whereas the negative predictive value of enhanced chest CT for the diagnosis of septated pleural effusion was 82.0% (95% CI 74.6–87.8). In order to compare the diagnostic efficacy of ultrasound and CT, we also described the subject operating curves (ROC curves) for both. As can be seen from Table 4, the diagnostic efficacy of ultrasound for septated pleural effusion was better than CT, with an area under the curve of up to 0.913. In contrast, the area under the curve for CT diagnosis was only 0.734.

**Table 3:** Diagnostic performance analysis of ultrasound/CT scan.

		Separated in MT No	Non- separated in MT No	Add up the total
Ultrasound	Separated	76	0	76
	Non-separated	16	193	209
Add up the total		92	193	285
CT scan	Separated	55	25	80
	Non-separated	37	168	205
Add up the total		92	193	285

**Table 4:** Estimates of the diagnostic test characteristics for ultrasound, chest CT, along with their 95% CIs.

	Ultrasound	CT scan
Sensitivity %, (95% CI)	82.6 (73.3–89.7)	59.8 (49.0–69.9)
Specificity %, (95% CI)	100.0 (98.1–NaN)	87.0 (81.5–91.4)
Positive predictive value %, (95% CI)	100.0 (95.3–100.0)	68.8 (59.0–77.4)
Negative predictive value %, (95% CI)	92.3 (87.5–NaN)	82.0 (74.6–87.8)
AUC (95% CI)	0.913 (0.874–0.952)	0.734 (0.678–0.790)

## Discussion

We posit that septated pleural effusion represents an intermediate stage in the progression of pleural effusion caused by diverse etiologies, including tumors, pleura infections, and immune reactions. This parallels the progression stages of complicated parapneumonic effusions (CPPEs), evolving from plasma exudate to fibrinous exudate, subsequent fibrous streak formation, culminating in pyothorax^1,13^. Patients with septated pleural effusions often encounter increased challenges in pleural fluid drainage compared to those with non-septated effusions, contributing to a higher likelihood of prolonged disease duration^3^. However, there is currently insufficient research and specific guidelines addressing septated pleural effusions. Additionally, there is a need to explore the utilization of ancillary screening methods for identifying septated pleural effusion^3^. Standardizing targeted treatment approaches for septated pleural effusion is equally imperative and requires dedicated investigation and evidence-based protocols. In this study, for the first time, we compared chest ultrasound and CT to find out the diagnostic value of different methods in recognizing pleural effusion septations.

In the method section of our article, we introduced a definition for septated pleural effusion. Specifically, individuals displaying multilocular septation are classified as having septated pleural effusions. Namely, septated pleural effusion refers to the formation of fibrous bands within the pleural effusion, forming multiple small cavernous compartments, which results in multiple septated cavities. This definition provides clarity on the distinction from loculated fluid collections. Loculated fluid collections refer to a condition where the fluid is localized to a specific region within the chest cavity, segregated from the surrounding areas and lacking communication with the rest of the chest^6^. Additionally, we conducted a retrospective comparison of the diagnostic efficacy between chest ultrasound and enhanced chest CT examination in cases of septated pleural effusions. Previous study has suggested that thoracic ultrasound exhibits better proficiency in discerning the internal structure of pleural effusion compared to enhanced chest CT^8,14^. Consequently, it has been considered superior to enhanced chest CT in determining septations^6,15,16^. While several studies have established the superior efficacy of ultrasound in diagnosing pleural adhesions compared to enhanced chest CT scanning, demonstrating sensitivities ranging from 80.6 to 88% and specificities from 81 to 96.1%^17–19^, there remains a paucity of research investigating the diagnostic effectiveness of ultrasound versus enhanced chest CT in identifying septated pleural effusions. Compared with other studies, our study used manifestations observed through thoracoscopy as the gold standard for diagnosing septated pleural effusion, and utilized septal and bubble signs as the criteria for diagnosing septated pleural effusion through CT and relied on chest ultrasound to identify septated pleural effusion through evident septal manifestations. Then, we compare the diagnostic efficacy of CT and ultrasound in identifying septated pleural effusion.

According to our findings, thoracic ultrasound exhibits superior sensitivity and specificity compared to enhanced chest CT in diagnosing septated pleural effusions, which is consistent with the findings of previous studies^6,7,20,21^. Therefore, chest ultrasound is highly recommended as an adjunct for preoperatively determining septated pleural effusion. However, as indicated in Table 4, the sensitivity of thoracic ultrasound is observed to be relatively low. Upon analysis, we attribute the lower sensitivity to our initial classification, where patients exhibiting the fourth ultrasound manifestation were categorized into the group without septated pleural effusion, and this group of patients were mostly patients with pyothorax or hemothorax, with 9 cases specifically identified as pyothorax. In their thoracic ultrasound examinations, a consistent observation of homogeneous hyperechoic signals (type 4) was noted. However, upon thoracoscopic examination, the actual presentation revealed gelatinous pus distributed amidst multicompartmental septations. Thus, the inclusion of this group of patients was a major contributor to the decreased sensitivity of ultrasound for detecting septation of pleural effusions. In reality, almost half of the patients displaying the fourth ultrasound presentation were later diagnosed with septated pleural effusion by medical thoracoscopy. Hence, we deduce that thoracic ultrasound exhibits notably high specificity and a certain level of sensitivity in detecting septated pleural effusion, surpassing the capabilities of thoracic CT. And when confronted with an ultrasound image portraying type 4 presentation of pleural effusion, the possibility that there may be a pleural effusion septation needs to be taken into account.

The primary limitation of this article lies in its retrospective nature. Consequently, we faced constraints in capturing an exhaustive array of imaging features from both ultrasound and enhanced chest CT. And due to the limited number of features we collected, we were unable to systematically select and identify the imaging characteristics that best reflect septated pleural effusion. Prospective studies are needed to further investigate the best ancillary tests for diagnosing septated pleural effusions and the corresponding imaging manifestations.

## Data Availability

The datasets used and/or analysed in the present study are available from the corresponding author on reasonable request.

## Abbreviations

CT: Computed tomography
ROC curves: Subject operating curves
CPPEs: Complicated parapneumonic effusions
